# A Short-Term Assessment of Nascent HIV-1 Transmission Clusters Among Newly Diagnosed Individuals Using Envelope Sequence-Based Phylogenetic Analyses

**DOI:** 10.1089/aid.2019.0142

**Published:** 2019-10-01

**Authors:** Alexis Kafando, Bouchra Serhir, Florence Doualla-Bell, Eric Fournier, Mohamed Ndongo Sangaré, Christine Martineau, Mohamed Sylla, Annie Chamberland, Mohamed El-Far, Hugues Charest, Cécile L. Tremblay

**Affiliations:** ^1^ Département de Microbiologie, Infectiologie et Immunologie, Faculté de Médecine, Université de Montréal, Montréal, Canada.; ^2^ Laboratoire de Santé Publique du Québec, Institut National de Santé publique du Québec, Sainte-Anne-de-Bellevue, Canada.; ^3^ Département de Médecine Sociale et Préventive, École de Santé Publique, Université de Montréal, Montréal, Canada.; ^4^ Centre de Recherche du Centre Hospitalier de l'Université de Montréal (CRCHUM), Montréal, Canada.

**Keywords:** HIV-1 transmission networks, clusters, envelope gene sequences, pairwise genetic distance

## Abstract

The identification of transmission clusters (TCs) of HIV-1 using phylogenetic analyses can provide insights into viral transmission network and help improve prevention strategies. We compared the use of partial HIV-1 envelope fragment of 1,070 bp with its loop 3 (108 bp) to determine its utility in inferring HIV-1 transmission clustering. Serum samples of recently (*n* = 106) and chronically (*n* = 156) HIV-1-infected patients with status confirmed were sequenced. HIV-1 envelope nucleotide-based phylogenetic analyses were used to infer HIV-1 TCs. Those were constructed using ClusterPickerGUI_1.2.3 considering a pairwise genetic distance of ≤10% threshold. Logistic regression analyses were used to examine the relationship between the demographic factors that were likely associated with HIV-1 clustering. Ninety-eight distinct consensus envelope sequences were subjected to phylogenetic analyses. Using a partial envelope fragment sequence, 42 sequences were grouped into 15 distinct small TCs while the V3 loop reproduces 10 clusters. The agreement between the partial envelope and the V3 loop fragments was significantly moderate with a Cohen's kappa (κ) coefficient of 0.59, *p* < .00001. The mean age (<38.8 years) and HIV-1 B subtype are two factors identified that were significantly associated with HIV-1 transmission clustering in the cohort, odds ratio (OR) = 0.25, 95% confidence interval (CI, 0.04–0.66), *p* = .002 and OR: 0.17, 95% CI (0.10–0.61), *p* = .011, respectively. The present study confirms that a partial fragment of the HIV-1 envelope sequence is a better predictor of transmission clustering. However, the loop 3 segment may be useful in screening purposes and may be more amenable to integration in surveillance programs.

## Introduction

In 2015, a total of 609 HIV cases were reported to the Public Health Laboratory in Quebec (LSPQ), including 299 new diagnoses.^1^ To better characterize transmission patterns,^2,3^ we evaluated molecular epidemiology methods using phylogenetic analyses of HIV genome sequences obtained from infected individuals. Pairwise genetic distances (PWDs) derived from *env*, *gag*, and *pol* nucleotide sequences were used to assess the relationship between sequences and therefore define study HIV transmission dynamics.^4,5^ Combining sequence-based clustering with risk factors and sociodemographic, clinical, and geographical parameters may help identify patterns of transmission in the HIV-infected population.^6–11^

The *env* gene, encoding glycoproteins gp120 and gp41, is the most variable region of the HIV-1 genome.^12–14^ GP120 contains five hypervariable regions (V1 to V5) and five conserved regions (C1–C5).^15–18^ The gp41 region consists of three domains, the ectodomain (ECD), the transmembrane domain, and the long cytoplasmic domain.^19^ Each subregion and each domain of the *env* play a key role in HIV pathogenesis.^16,18,20^

In the literature, *pol* gene-derived sequences are mostly used for phylogenetic analysis to infer HIV-1 transmission clustering.^4^ It is readily available in clinical laboratories performing drug resistance testing in most countries. However, little is known about the potential of HIV-1 envelope-derived sequences for this application.^21,22^ It has been shown that within-host *env* genetic diversity is significantly associated with HIV-1 Fiebig stage and disease progression.^23,24^ The HIV-1 evolution in a new recipient may recover some ancestral features of infected donors,^25^ such as the genetic distance between the donor and recipient.^21,26^ Such characteristics may be used to establish the closely transmitted network (cluster) between HIV-infected individuals.

The first objective of this study was to assess the potential of HIV *env*-derived sequences (1,070 bp) to determine HIV-1 transmission clustering. The second objective was to investigate whether shorter portions of the *env* gene, such as the *env* V3 loop-derived sequences (108 bp), could be a valuable tool to achieve the same mean while reducing technical, time, and cost constraints^27,28^ associated with partial but long-length *env* sequencing. The third objective was to identify risk factors associated with HIV transmission clustering.

## Materials and Methods

### Patients and specimens

Serum samples that were reactive in Quebec diagnostic laboratories using a screening HIV 1/2 immunoassay (EIA) were submitted to the LSPQ for confirmation by Western blot (WB) and/or p24 EIA. Positive p24 antigen samples were *de facto* classified as recent infections. Samples from newly confirmed HIV-1 individuals by WB were then submitted to a recent infection testing algorithm (RITA) based on antibody avidity. The latter combines a Centers for Disease Control and Prevention (CDC)-modified Bio-Rad Avidity Assay and a Sedia-LAg-Avidity assay.^29^ Recent infections were defined as a sample that was positive for HIV-1 p24 antigen or positive for HIV-1 antibodies by WB but classified as “recent” by RITA (≤136 days of infection). Established infections were defined as samples positive by WB and classified as long-standing by RITA (>136 days of infection).

Based on these criteria, we selected 262 newly diagnosed HIV samples that included recent (*n* = 106) and long-standing (*n* = 156) infections collected in 2015. The risk factors and clinical and epidemiological parameter data reported for each sample were extracted from the HIV provincial surveillance program.

### Amplification and sequencing of partial envelope fragment

Amplification and sequencing protocols were conducted as previously described by Kafando *et al.*^30^ Briefly, total nucleic acids were extracted from 100 μL of serum using an automated BioRobot MDx extraction platform. HIV-1 RNA was amplified using the Superscript III One-Step RT-PCR system with Platinum^®^ Taq DNA polymerase (Invitrogen, Thermo Fisher Scientific, and Carlsbad, CA) with primers *env-up* forward (5′-GTTTCTTTTAGGCATCTCCTATGGCAGGAAGAAG-3′, HXB2 positions 5957–5983) and *env-lo* reverse (5′-GTTTCTTCCAGTCCCCCCTTTTCTTTTAAAAAG-3′, HXB2 positions 9063–9088).^31^ Nested amplification was performed using the Expand™ High Fidelity PCR System Kit (Roche Diagnostics, Indianapolis). Primers E60F forward (5′-TAATCAGTTTATGGGATCAAAGC-3′, HXB2 positions 6547–6569)^32^ and E55R reverse (5′-GCCCCAGACTGTGAGTTGCAACAGATG-3′, HXB2 positions 7940–7914),^33^ were used, generating PCR products covering ≈1,400 bp of the *env* gene. Amplification conditions, library preparation, and Illumina MiSeq next-generation sequencing (NGS) were conducted as previously described.^30^ Following quality control with FastQC (http://www.bioinformatics.babraham.ac.uk/projects/fastqc), raw sequence reads were *de novo* assembled using the Iterative Virus Assembler (IVA)^34^ to generate a consensus sequence.

### Sequence data processing, phylogenetic analysis, and HIV transmission cluster reconstruction

All consensus *env* nucleic acid sequences were aligned with molecular evolutionary genetic analysis, using MEGA7 software (www.megasoftware.net) under ClustalW methods.^35^ All aligned sequences were submitted to MAFFT multiple sequence alignment software version 7^36^ to verify the reliability of the alignments. The human immunodeficiency virus type 1 K03455.1 (HXB2) *env* nucleotide sequence (nt) positions (6225 to 8795) were included in the alignment to serve as a reference. Matching sequences, excluding gaps with equal lengths (HXB2 nt positions 6831 to 7900 ≈ 1,070 bp) in this case, were selected for phylogenetic analyses. The *env* partial fragment analyzed included the gp120-C2 to C5 subregions (HXB2 nt positions 6813 to 7757) and the gp41 partial ECD (HXB2 nt positions 7758 to 7915). The HIV-1 *env* gp120-V3 loop sequences (HXB2 genome nt positions 7110 to 7217 ≈ 108 bp) were also analyzed separately. Phylogenetic trees were constructed in MEGA7 using the maximum likelihood algorithm, and their reliability was estimated from 1,000 bootstrap replicates. Transmission clusters (TCs) were evaluated among sequences that grouped around common proximal nodes with ≥99% bootstrap, as supported by a previous study.^11^ In the present study, in the absence of the gold standard for HIV-1 envelope sequence-based clustering, we considered a PWD of ≤10% as a threshold, as suggested by Novitsky *et al.*^21^ The HIV-1 transmission clustering was defined as two or more HIV-1-infected individual genomic sequences whose branches were grouped under a genetic distance threshold in the phylogenetic tree.^4^ The interpretation of the extent of the relevant transmission clustering depended on the genetic distance threshold, the time that it was established, the number sequence data set, the sampling densities, the node bootstrap, and the HIV-1 genomic region concern.^4,21^ We considered a high PWD (≤10%) to define clustering for the HIV-1 envelope because it presented the highest variability compared with *GAG* and *Pol* (PWD ≤1.5%). The extent of the HIV TC was classified as unique (1 member), small (2–4 members), or large (5–60 members) in the transmission chain.^6^ We used ClusterPickerGUI_1.2.3 (http://hiv.bio.ed.ac.uk/software.html) to construct cluster trees^37^ and FigTree v1.4.3 (http://tree.bio.ed.ac.uk/software/figtree/) to view them. We used Dendroscope version 3.5.10 to build a Tanglegram of connected taxa between rooted phylogenetic trees and networks.^38,39^ The NCBI subtyping tool^40^ was used to determine HIV viral subtypes, which was confirmed by the REGA HIV-1 subtyping tool version 3.^41^

### Statistical analyses

We used bivariate and multivariate analyses to determine the independence of associations between exposed variables (epidemiologic and clinical factors) and outcome (clustering). Categorical variables were compared using a chi-square or Fisher's exact test, and continuous variables were compared using a two-sample Student's *t*-test or Wilcoxon rank sum test. All variables with *p* < .25 as a cutoff point from bivariate analysis were included in the multivariate analysis using logistic regression. At this point, *p* < .05^42,43^ using the Wald test was considered to be statistically significant, and selected variables were included in the final regression model. Statistics analyses were performed in SPSS version 24 and STATA version 14. The determination of agreement between *env* gp120-V3 sequence clusterings determining with a partial *env* fragment as a gold standard to correctly identify individuals in a cluster tree was assessed using Cohen's kappa coefficient. The estimated parameters were sensitivity, specificity, positive predictive value, and negative predictive value. Stata IC version 14 was used as a statistical package, and a *p* value less than 5% was considered statistically significant.

### Ethics statement

All work was conducted in accordance with the Declaration of Helsinki in terms of informed consent. All samples were anonymized before we accessed them for this study. No nominal information was used for analysis or data management. Ethical approval was given and renewed yearly by our Institutional Review Board (IRB): the “Comité d'éthique et de la recherche des Centres hospitaliers affiliés à l'Université de Montréal”.

## Results

**FIG. 1.**
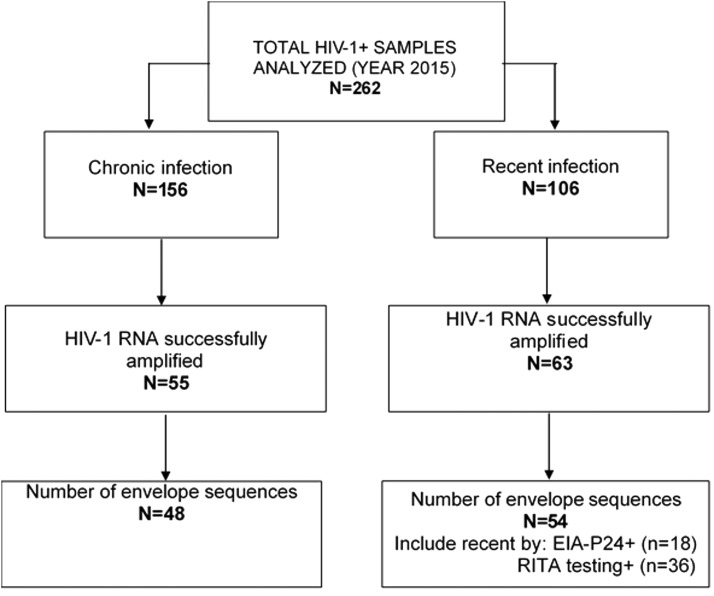
Flowchart of HIV-1 envelope sequences used in this study. The figure presents the total number of specimens sampled (*n* = 262) and the final sequences obtained after amplification, sequencing, and sequence analysis processes (*n* = 102). EIA, enzyme immunoassay; RITA, recent infection testing algorithm.

Amplification and DNA sequencing of HIV-1 *env* were attempted on 262 specimens and were successful for 39% of them (*n* = 102) (Fig. 1). This moderate level of amplification success could be mediated by multiple factors affecting the specimen quality such as the long-term storage of serum, viral RNA extraction procedures and enzymes used in the PCR amplification as well as the viral loads of infected individuals (VL < 20,000 copies/ml). In addition, depending on the length the HIV-1 envelope sequence to be amplified, this procedure is known to be challenging.^27,28^

Of the included HIV-1 *env* sequences (*n* = 102), 47% (*n* = 48) and 35% (*n* = 36) were classified as chronic and recent HIV infections, respectively, using the RITA (26), whereas 18% (*n* = 18) were found to be recent as confirmed by EIA-p24 testing (Fig. 1). The demographic characteristics are described in Table 1. Briefly, 34% of individuals were asymptomatic, whereas 13% of individuals presented acute HIV infection symptoms, and 11% had reached the AIDS stage (Table 1). The mean CD4 count in total population was 375 cell/mm^3^, range [5–1,150]. The mean HIV-1 viral load in total population was 4.94 log_10_ copies/ mL, range [1.60–8.20], and the mean age was 38.8 years, range [18–78]. Of the 102 individuals, 79.4% were infected with HIV-1 subtypes B, and 20.6% with HIV-1 non-B subtypes. Age and baseline CD4^+^ T cell counts were significantly different between the recent and chronic infections using the Wilcoxon rank sum test. The mean age of recently infected individuals was 34.15, range [18–78], and for chronic infections, it was 44.03, range [21–58], *p* = .0002. The mean baseline CD4^+^ T cell count was 521, range [12–1,150] in recent infections and 186, range [5–698] in chronic infections.

**Table 1.** Population Characteristics

*Variable characteristics*	*Recent*	*Chronic*	*Total*
Gender, *n* (% of individuals)			
Female	1 (1.90)	13 (28.20)	14 (14.14)
Male	52 (98.10)	33 (71.80)	85 (85.86)
Risk factor for HIV acquisition, *n* (% of individuals)			
MSM	36 (85.71)	19 (48.72)	55 (67.90)
Heterosexual	0	3 (7.69)	3 (3.70)
Relationship with at-risk heterosexual	2 (4.76)	2 (5.13)	4 (4.94)
Endemic country	1 (2.38)	13 (33.33)	14 (17.28)
MSM/IDU	3 (7.14)	0	3 (3.70)
IDU	0	2 (5.13)	2 (2.47)
Place of birth, *n* (% of individuals)			
Canadian	27 (65.85)	19 (48.72)	46 (57.50)
Caribbean	3 (7.32)	6 (15.38)	9 (11.25)
Europe	5 (12.20)	1 (2.56)	6 (7.50)
North Africa and Middle East	1 (2.44)	0	1 (1.25)
Sub-Saharan Africa	2 (4.83)	10 (25.64)	12 (15)
Latin, Central, and South America	4.88	2 (5.13)	4 (5)
Asia	1 (2.44)	0	1 (1.25)
Aboriginal (First Nations)	0	1 (2.56)	1 (1.25)
HIV subtype, *n* (% of individuals)			
B	45 (83.30)	36 (75.00)	81 (79.40)
Non-B	9 (17.70)	12 (25.00)	21 (20.60)
Age, *n* [mean; range]	31 [18–78]	46.5 [21–77]	37 [18–78]
15–24 years	12	2	14
25–34 years	23	11	34
35–44 years	6	7	13
45–54 years	7	19	26
55–64 years	4	5	9
>64 years	1	2	3
Baseline CD4^+^ T cell count (cells/mm^3^), no. of individuals [median; range]	42 [458.5; 12–1,150]	36 [175; 5–698]	78 [375; 5–1,150]
>500	19 [710; 506–1,150]	3 [609; 580–698]	22 [700; 506–1,150)
351–500	11 [415; 390–490]	7 [420; 360–485]	18 [417.5;360–490)
201–350	8 [250; 217–320]	5 [302; 220–340]	13 [252; 217–340)
≤200	4 [185; 12–200]	21 [59; 5–196]	25 [63; 5–200]
HIV viral load (log10 copy/mL), no. of individuals [median; range]	42 [4.94; 3.43–8.20]	38 [4.76; 1.60–6.34]	80 [4.90;1.60–8.20]
>5	20 [5.53; 5–8.20]	15 [5.60; 5.15–6.34]	35 [5.60; 5–8.20]
<5	22 [4.46; 3.43–.96]	23 [4.25; 1.60–4.98]	45[4.27;1.60–4.98]

Table presents the repartition of demographic, epidemiologic, clinical, and risk factors of the study population by infection status (recent or chronic).

IDU, injecting drug user; MSM, men who have sex with men.

### HIV-1 partial envelope fragment-length sequence-based clustering using phylogenetic analysis

**FIG. 2.**
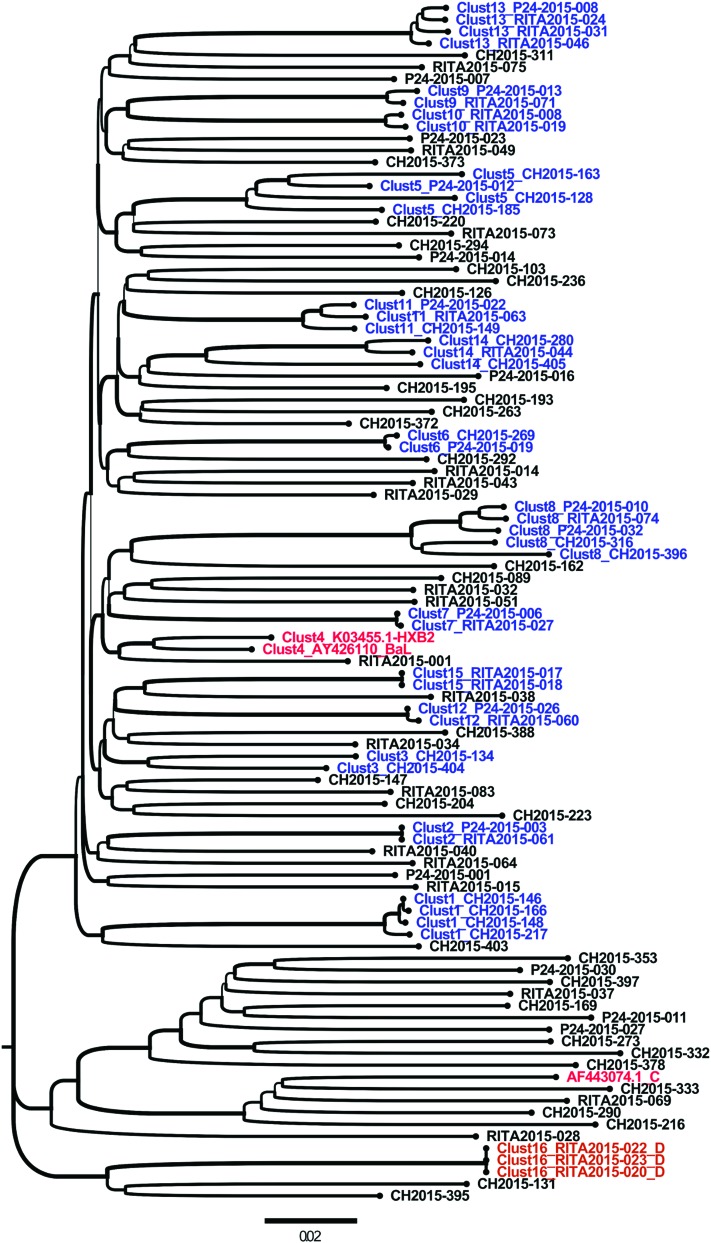
Molecular phylogenetic reconstruction of HIV-1 TCs among newly and chronically HIV-infected individuals in 2015 in Quebec using partial *env-*derived sequences (1,070 bp). The cluster tree contains 101 sequences from HIV-1-infected individuals, including 98 from the study cohort and 3 reference sequences introduced in the analyses as controls (*AF443074.1_C*, *AY426110_BaL*, and *K03455.1-HXB2*). A total of 42 HIV-1 individuals' sequences are grouped into 16 distinct small HIV-1 TCs. These clusters are labeled from Clust1 to Clust16 and are depicted using different colors. The cohort-derived clusters are shown in *blue* for subtype B (Clust1 to Clust15, except Clust4) and *orange* for subtype D (Clust16). The control sequence names are shown in *red* (Clust4). Sequences that did not form a cluster are shown in *black*. Each depicted cluster satisfied ≥99% support on bootstrap resampling of 1,000 replicates and 10% of pairwise distance.^21^ CH, chronic HIV-1 infection sequences; p24, recent infection status determined by p24 antigen positivity; RITA, recent infection status determined by RITA^29^; TCs, transmission clusters. Color images are available online.

From the 102 sequences analyzed, 4 were excluded because of insufficient *env* length. Finally, 98 sequences with satisfactory and similar lengths were included in the phylogenetic analysis. Of these 98 sequences, 57.14% (*n* = 56) did not form clusters, whereas 42.85% (*n* = 42) were part of 16 small clusters ranging from two to five individuals using the partial *env* fragment length (1,070 bp) at a distance cutoff of 0.1 (Fig. 2). The HIV-1 subtype B envelope sequence represented 93.3% of clusters identified (*n* = 14/15) and were labeled Clust1 to 15, except for Clust4 that was constituted by HXB2 and BAL reference sequences. The non-B HIV-1 subtypes, which represented 7.14% and 6.7% (*n* = 1/15), labeled Clust16, are shown in Figure 2.

Sequences from recently HIV-1-infected individuals were mostly represented in clusters; 50% were (*n* = 27/54) involved, whereas 31.21% (*n* = 15/48) comprised chronic infections (Fig. 2). The inclusion or noninclusion in clusters for acutely and chronically HIV-1-infected individual envelope sequences was statistically significant using the chi-square test as follows: odds ratio (OR): 0.4, *p* = .02.

### HIV-1 envelope gp120 loop 3 (V3) sequence-based clustering using phylogenetic analysis

**FIG. 3.**
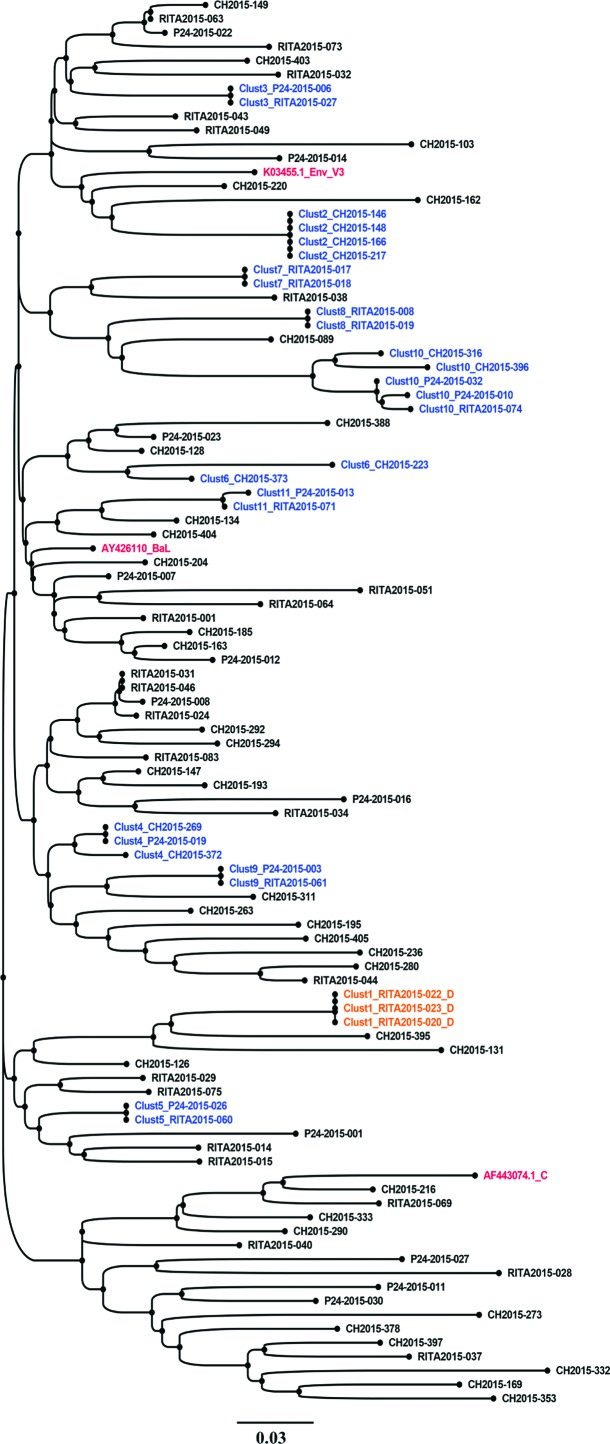
Molecular phylogenetic reconstruction of HIV-1 TCs among newly and chronically HIV-infected individuals using *env* gp120 V3 loop-derived sequences (108 bp). The cluster tree contains 101 sequences from HIV-1-infected individual's *env*-V3 sequences, including 98 from the study cohort and 3 reference sequences introduced in the analyses as controls (AF443074.1_C, AY426110_BaL, and K03455.1-HXB2). Each tip represents an individual patient. A total of 31 HIV-1 sequences grouped into 11 distinct small HIV-1 TCs with two to five members per cluster. These clusters are labeled from Clust1 to Clust11 and are depicted using different colors. Clusters including sequence names are shown in *blue* for subtype B (Clust2 to Clust11) and *orange* for subtype D (Clust1). The control sequence names are shown in *red*. Sequences that did not cluster are shown in *black*. Each depicted cluster satisfied ≥99% support on bootstrap resampling of 1,000 replicates and 10% of pairwise distance.^21^ CH, chronic HIV-1 infection sequences; p24, recent infection status determined by p24 antigen positivity; RITA, recent infection status determined by RITA.^29^ Color images are available online.

Phylogenetic analyses using only the V3 loop (108 bp)-derived sequences were also performed to identify HIV-1 TCs. The *env* loop 3 sequence-based clustering reproduced 66.66% (*n* = 10/15) of the TCs observed with the partial *env* fragment length (Fig. 3). Five clusters labeled (Clust3, 5, 11, 13, and 14), shown in Figure 2, were not observed when using the *env* V3 sequence-based clustering (Fig. 3). However, it identified an additional cluster (Clust6) (Fig. 3) that was not detected by the partial *env* fragment length analyses.

### Agreement between the HIV-1 partial envelope fragment lengths of the V3 loop-derived sequences as independent tools to perform HIV-1 transmission clustering

**FIG. 4.**
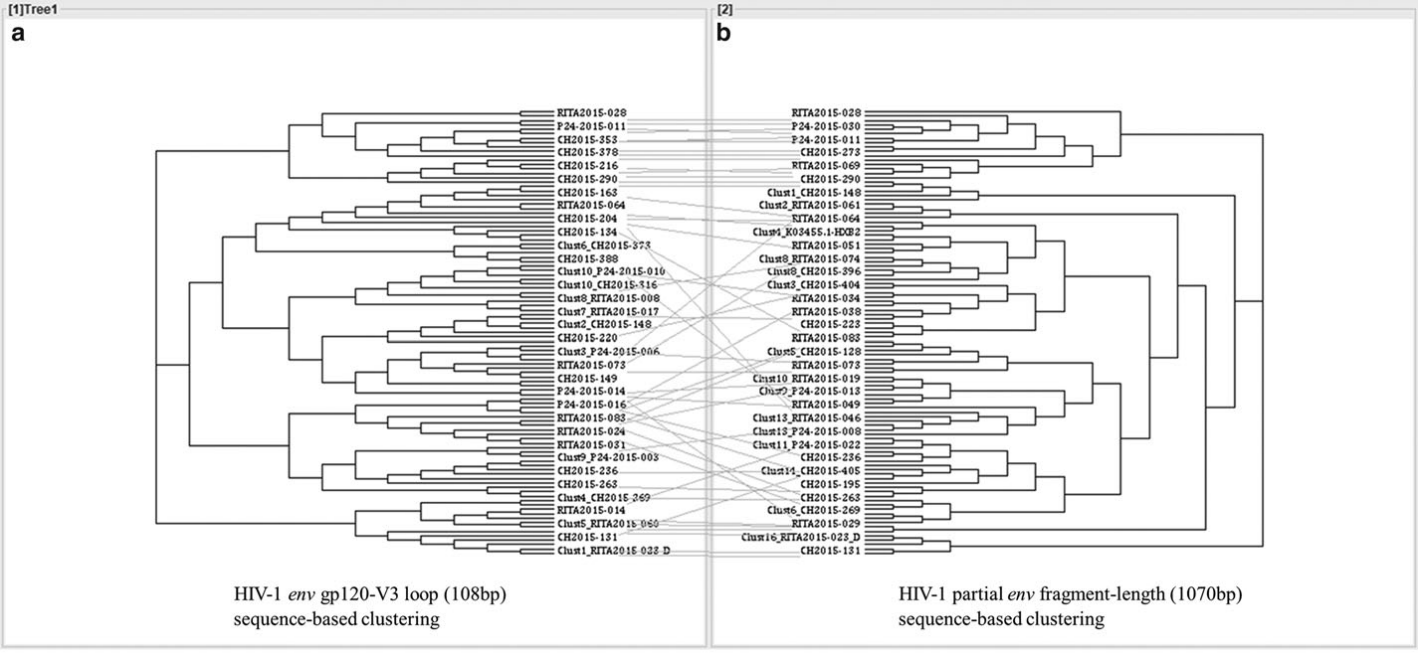
Tanglegrams of rooted phylogenetic trees and networks comparing the HIV-1 envelope gp120 loop 3 **(a)** with the HIV-1 partial envelope length **(b)** sequence-based clustering. The linked clusters and sequences between the two trees are connected by a *gray line*.

The *env*-V3 sequence-based clustering reproduced 10 of the 15 clusters previously identified with the *env* 1,070 bp sequence length. An additional cluster (C6) was identified only with *env*-V3 loop sequence-based clustering, shown in Figure 4.

The sensitivity, specificity, positive predictive value, and negative predictive value of *env* gp120-V3 in determining TCs were 70%, 98.6%, 95.5%, and 88.8%, respectively, compared with the partial *env* fragment length (1,070 bp)-derived sequences as the gold standard (Table 2). The concordance between the gold standard and *env* gp120 V3 cluster determination was assessed by Cohen's kappa coefficient and demonstrated a significantly moderate agreement, κ = 0.59, *p* < .00001 (Table 2).

**Table 2.** Performance of the *env* gp120-V3 Sequences in Determining HIV Transmission Clustering Compared with the Use of the Full *env* Fragment

*Accuracy parameters*	*Value % (*n/N*)*	*95% CI*
Sensitivity	61.9 (26/42)	45.6–76.4
Specificity	95.0 (57/60)	86.1–99
Positive predictive value	89.7 (26/29)	72.6–97.8
Negative predictive value	78.1 (57/73)	66.9–86.9

Table presents the performance of the env gp120-V3 sequence-based clustering (108 bp) using partial HIV-1 envelope fragment length (1,070 bp) as the gold standard. The sensitivity and specificity were 61.9% (45.6–76.4) and 95% (86.1–99), respectively. In addition, the positive predictive value and negative predictive value were 89.7% (72.6–97.8) and 78.1% (66.9–86.9), respectively. Furthermore, significant and moderate agreement was documented using Cohen's kappa coefficient (κ = 0.59, *p* < .00001).

CI, confidence interval; *n*, number of clusters identified by *env* gp120 V3 loop-derived sequences; *N*, number of clusters identified by the gold standard.

### Agreement between the partial HIV-1 envelope fragment sequence lengths with the gp120 C2V3C3 region-derived sequences as independent tools for identifying HIV-1 TCs

We also compared the partial *env* fragment length (1,070 bp) with the gp120 C2V3C3 regions (HXB2 nt positions 6813 to 7376 ≈ 564 bp) to improve the moderate sensitivity associated with the V3 sequence-based clustering identification. Surprisingly, the gp120 C2V3C3 regions identified only 6 of the 15 clusters (40%) observed using the partial *env* fragment sequence at PWD of ≤10%. This unexpected result represented less than the number of clusters identified by V3 loop-derived sequences alone, estimated to be 66.66% compared with the same gold standard.

### Description of factors associated with HIV transmission clustering

The recently HIV-1-infected subjects defined by RITA^29^ represented 53.4% of the TCs, followed by acutely infected individuals (EIA-p24+) at 23.3% and chronically infected individuals at 23.3% (Fig. 1 and Table 1). The HIV-1 subtypes B and D comprised 93% and 7% of clusters, respectively. The distribution of the HIV-1 TCs by epidemiologic, clinical, and risk factors of acquisition is presented in Table 3.

**Table 3.** Summary Statistics of the Distribution of HIV-1 Transmission Clusters by Factors

*Parameters*	*HIV transmission clustering*	
	*No (%)*	*Yes (%)*
Age		
<38	23 (39.66)	27 (65.85)
≥38	35 (60.34)	14 (34.15)
Gender		
Male	46 (79.31)	39 (95.12)
Female	12 (20.69)	2 (4.88)
HIV-subtype		
B	42 (70)	39 (92.86)
Non-B	18 (30)	3 (7.14)
Ethnicity		
White	34 (85)	25 (83,33)
Black	16 (4)	5 (16.16)
Motivation for HIV testing		
Symptomatic person	25 (50)	16 (51.61)
Screening in an asymptomatic person	17 (24)	13 (41.94)
Confirmation of previous positive test	8 (16)	2 (6.45)
HIV-1 clinical status		
Asymptomatic	19 (38)	15 (48.39)
Acute infection	18 (36)	12 (38.71)
Symptomatic infection + AIDS	13(26)	4 (12.90)
HIV transmission risk factors		
MSM	32 (64)	23 (74.19)
MSM/IDU+IDU	3 (6)	2 (6.45)
Heterosexual	15(30)	6 (19.35)
Log^10^ HIV viral load		
<5	29 (59.18)	15 (48.39)
≥5	20 (40.82)	16 (51.61)
CD4 count value		
<350	24 (51.06)	14 (45.16)
≥350	23 (48.94)	17 (54.84)
HIV infection status by test		
Chronic infection by RITA^29^	33 (55)	15 (35.71)
Acute/early infection by EIA-p24 testing	9 (15)	10 (23.81)
Recent by RITA^29^	18 (30)	17 (40.48)
HIV-1 infection status		
Recent (acute/early recent by RITA)	**27** (45)	**27** (64.29)
Chronic	**33** (55)	**15** (35.71)

*Bold* values illustrate the number of recent versus chronic HIV-1 infection sequences include or non-include in HIV-1 transmission clusters.

Table presents the summary statistics of the demographic, epidemiologic, clinical, and risk factors of HIV-1-infected individuals associated with inclusion in or not in clusters.

EIA, enzyme immunoassay; RITA, recent infection testing algorithm.

The mean age of HIV-1-infected individuals included in clusters was 33.34 years [18–58], the mean CD4 count was 390.22 cell/mm^3^ [10–871], and the mean HIV-1 viral load was 5.12 log^10^ copies/mL [1.60–8.20]. Mean age (<38.8 vs. ≥38.8 years) and HIV-1 subtype (B vs. non-B) were identified as two factors significantly associated with HIV-1 TCs: mean age (OR = 0.25, 95% confidence interval [CI, 0.04–0.66], *p* = .002) and HIV-1 subtype (OR = 0.17, 95% CI [0.10–0.61], *p* = .011) (Table 4).

**Table 4.** Factors Associated with HIV-1 Transmission Clustering Using Logistic Regression Analysis

*Factors*	*Bivariate analysis*		*Multivariate analysis*		
	n	p	*OR*	*95% CI*	P
Ethnicity					
Black	21	.74			NS
Nonblack	60	Ref.			
Gender					
Male	85	.041			NS
Female	14	Ref			
Age (mean)					**S**
<38.8 years	**52**	**.001**	**0.25**	**0.04–0.66**	**.002**
≥38.8 years	47	Ref.			
HIV transmission route					**NS**
MSM intercourse	55	.18			
Heterosexual intercourse	21	.83			
IDU and MSM/IDU intercourse	5	Ref.			
Motivation for HIV testing					**NS**
Screening in an asymptomatic person	30	.20			
Symptomatic person	41	.27			
Confirmation of previous positive test	10	Ref.			
Clinical status					**NS**
Acute infections	13	.94			
Chronic infection symptoms	6	.18			
Nonspecific diseases and symptoms	17	.38			
AIDS	11	.23			
Asymptomatic	34	Ref.			
HIV subtype					**S**
B	81	**.009**	**0.17**	**0.10–0.61**	**.011**
Non-B	**21**	Ref.			
HIV-1 infection status					**NS**
Recent by RITA^29^ + recent by EIA-p24+	54	.057			
chronic by RITA^29^	48	Ref			
CD4 cell count (cells/mm^3^)					**NS**
≥500	22	.895			
<500	56	Ref			
HIV-1 viral load (copies/mL)					**NS**
≥100,000	36	.345			
<100,000	44	Ref.			
HIV-1 viral load (copies/mL)					**NS**
≥1,500	77	.337			
<1,500	3	Ref			
VL_log10					NS
>5	36	.345			
<5	44	Ref			

Table presents results of logistic regression analysis, including bivariate analysis showing that the HIV-1 subtype, gender, and mean age of infected individuals were statistically significant (*p* < .05). However, all factors with a *p* value ≤.25 were subjected to multivariate analysis. For multivariate analysis, HIV-1 subtype (B vs. non-B), OR = 0.25 (0.04–0.66), *p* = .002, and patient mean age (<38.8 years compared with those aged ≥38.8 years), OR = 0.17 (0.10–0.61), *p* = .011, constitute two factors significantly associated with HIV-1 transmission clustering in this study population.

*Bold* values illustrate statistically significant results.

Ref: variable of comparison. The statistically significant variables were indicated by their *p* values.

NS, statistically nonsignificant; OR, odds ratio; S, statistically significant.

## Discussion

HIV-1 envelope sequence inference was used to reveal HIV-1 transmission clustering (network) among newly diagnosed individuals in 2015 in Quebec, Canada. The first analyses used a partial *env* fragment length (1,070 bp) that identified 15 small TCs, including 42 HIV-infected individuals. Using only the *env* gp120 V3 sequences (108 bp), we identified 11 small clusters, thus reproducing 66.7% (10/15) of clusters that were previously detected by the partial- *env* fragment sequence length, which have been considered as the gold standard. We used the envelope loop 3 (V3) fragment as comparison because it is the most conserved of the HIV-1 *env* hypervariable regions, and its sequences are frequently used to predict coreceptor tropism^44–50^ and may be available for intention-to-treat analysis using entry inhibitors in clinics.

The agreement between these two approaches was moderate (Cohen's kappa coefficient (κ) = 0.59). We tested different cutoff values of V3 sequences (1% to 20%) compared with partial *env* fragment sequences, but they did not enhance the accuracy of the agreement of the approaches. This observation underlies and confirms that the length of HIV-1 genome sequences analyzed inflects on TC determination.^26^ Therefore, using only *env* V3 loop sequences may underestimate HIV-1 TCs.

Although we included two constant regions of the gp120 (C2, C3) with the V3 loop and compared with the HIV-1 partial *env* fragment in the hope of increasing the degree of sensitivity of a number of cluster estimates, unexpectedly, the sequence derived from the gp120_C2-V3-C3 regions identified only 40% of the clusters, which is lesser than the number of clusters identified by the sequences derived from the V3 loop alone (66.66%) compared with the HIV-1 partial *env* fragment. The three regions decreased the sensitivity associated with the use of a larger fragment comprising two constant regions and the V3 loop. It may be that not all have a good degree of conservation of the nucleotide sequences. The nucleotide sequence diversity introduced by the two constant regions (C2 and C3) may have contributed to increasing the genetic distance threshold between individuals up to >10–15%, contributing to reduce the sensitivity of the estimated number of clusters. We believe that increasing the length of the sequence up to 524 bp, including only constant regions (C2, C3 with V3), may not increase the degree of sensitivity of cluster estimates compared with partial *env* fragment length.

However, the small number of *env* sequence data sets used in this study did not yield formal conclusions. Further studies including a large *env* sequence data set and the combination of different *env* segments can help confirm the present result.

We have proceeded to new phylogenetic analyses concerning the gp120 C2V3C3 clustering and reached the same conclusions. We will consider in the future a large sequence data set and may evaluate different segments of the envelope sequence.

However, in screening for public health surveillance purposes, it may be useful as V3 loop sequencing techniques are routinely performed in clinical laboratories to inform virus tropism and the use of CCR5 inhibitors in treatment. A recent study has shown that the V3 loop is one of most predictable segments of the HIV-1 envelope and that using its sequences contributes to estimating the recency of an infection.^30^ Hence, it may also be useful for real-time HIV-1 TC detection in complementing existing methods.

Previous studies have already demonstrated that the near full-length genome sequences (9,719 bp) of HIV-1 are the best tool for estimating the extent of HIV clusters.^26,51^ However, the methodological and cost constraints associated with full-genome sequencing preclude its use in routine clinical epidemiology studies. The estimate of HIV TCs (size and extent) depends on the selected gene and sequence length.^21^ It may negatively (underestimation) or positively (overestimation) affect the following: the cutoff value defined as genetic distance,^21^ the type of sequencing (Sanger or NGS), the number of sequences in data sets, the number of variable sites or the period covered by the study,^4,26^ the timely availability of sequence data,^52^ and the molecular phylogenetic methods used for transmission chain defining.^4,53,54^ This study covered a 1-year period (2015) that may underestimate transmission events occurring outside of this period. If sequencing is performed on a regular basis, it may help track the early founder of nascent clusters before the growth that will be identified in the following periods and help adapt prevention strategies for at-risk populations. Considering CDC Guide, June 2018, the identification of most linked sequences over a short period of time could possibly indicate that transmission occurs rapidly within a group.^55^

The cutoff value of the PWD used for this study was ≤10%, as proposed by Novitsky *et al.*^21^ We considered these cutoff values because they are not a gold standard concerning HIV envelope sequence-based clustering, and the study by Novitsky *et al.*^21^ is the most recent and the method used (PWD) to define clustering was adapted to the present study than others. Using a small cutoff value may underestimate cluster sizes and, on the contrary, using a large cutoff value may overestimate them. Therefore, the cutoff value has to be well defined^5,8,21,56,57^ according to the study by Novitsky *et al.*^21^ Specially, in considering the specificity of each the different HIV-1 subtypes and CRFs, it may be also important to determine the best cutoff value when using the full-length HIV-1 envelope fragment (GP160) or its specific subregions or domain (GP120, GP41)-derived sequences for cluster estimates and established correlation between these tools. Further studies that may also evaluate the performance of using the near full-length HIV-1 genome and its three regions (*GAG*, *Pol*, and *Env*) adjusted by subtypes as independent tools for determining HIV-1 TCs are also encouraged using large sequence data sets.

Compared with earlier studies conducted by Brenner *et al.*^8,58,59^ in Québec for longer periods of time and using a large sequence data set of the HIV-1 *pol* region (*n* = 1,277), 30 large TCs (20+/cluster) occurring over 13 years (2002 to 2015)^59^ were identified. Lubelchek *et al.* also conducted a similar study in Chicago using a large data set of *pol* gene sequences and identified a single large TC in a total of 26 clusters observed.^11^ Many HIV-1 TC studies have used *pol* gene sequences as these sequences are often available from clinical laboratories performing drug resistance testing (53). These findings contrast those of the present study, where no large cluster has been identified. The reason for the difference may be due to the limited *env* sequence data used in this study (*n* = 102) and the restricted period (1 year).^8,11,58,59^ Nevertheless, for this 1-year period, we were able to identify and highlight the existence of HIV-1 clustering between newly HIV-1-diagnosed patients. The present study aimed to track the nascent or forming clusters in real time to help adapt early prevention strategies that may limit the formation of large clusters.

Bivariate analysis identified that newly HIV-1-infected individuals are significantly more associated with HIV-1 transmission clustering than chronically infected individuals, OR = 2.20, *p* = .04, using Fisher's exact test. This observation is in agreement with results presented by Brenner *et al.*,^59^ Dennis *et al.*,^52,60^ and Miller *et al.*^61^ The results may be explained by the extreme fitness early founder viruses that successfully establish infections^62–64^ and the higher viral load during acute infection.

Multivariate analyses identified two factors that were likely associated with TCs: the mean age (<38.8 years) and the HIV-1 B subtype. Individuals younger than 38.8 years were more likely to form TCs than those older than 38.8 years, OR = 3.01, *p* = .001 (bivariate analysis), and OR = 0.25, *p* = .002 (multivariate analysis). This result reflects the profile of the HIV epidemics in Quebec, Canada, where individuals aged between 30 and 39 years accounted for 28% of reported cases in 2016.^65^ Individuals infected by the HIV-1 B subtype were more likely to form clusters (OR = 0.17) than non-B subtypes, OR = 5.57, *p* = .009 (bivariate analysis), and OR = .0.17, *p* = .01 (multivariate analysis). This finding also reflects the most prevalent subtype circulating in Quebec. The HIV-infected men who have sex with men (MSM) population is generally infected by HIV-1 B viruses and represents a large proportion of new cases in Quebec (67.90%).^65^

The objective of the present study was to evaluate tools to detect newly HIV-infected individuals who have the potential to transmit and sustain HIV epidemics as early as possible. Newly HIV-infected individuals are generally unaware of their infection status^66,67^ and therefore can contribute to the spread of infection.^61,68^ High viral loads observed during acute infection^69–71^ also constitute a factor that enhances HIV transmission of newly HIV-infected individuals. HIV-1 viral load (>10,000 copies/mL) and CD4 counts >350 generally constitute biological factors significantly associated with HIV-1 transmission clustering.^57^ Our study did not find a significant association of viral load or CD4 counts with clustering. The difference may be due to the fact that only 23% of clusters occurred in individuals with acute infection; therefore, all other clusters occurred in individuals with dampened viral load (chronic or recent infection <6 months). The timing of viral load assessment after diagnostic testing might also be a factor. Bivariate analysis identified gender (male) as significantly associated with TCs, OR = 5.08, *p* = .02. This reflects the HIV epidemic profile in Québec, with MSM being the highest at-risk group.^1^ In contrast to studies conducted in the United States by Lubelchek *et al.*^11^ and Paz-Bailey *et al.*,^72^ the black race was not significantly associated with clusters in the present study. This finding reflects the demographics of the HIV epidemic of Quebec, where black people are not overrepresented.^1,65^ In general, the results of this study suggest the importance of real-time follow-up of transmission networks regarding the early clusters of transmission established in a single year from newly HIV-infected individuals. The short-term assessment of early cluster building may help improve quick responses to prevent HIV transmission by identifying the nascent or forming clusters and populations at high risk.

## Conclusion

Our results confirm the presence of small HIV TCs among HIV-1-infected individuals in Quebec. HIV subtype B-infected individuals and individuals younger than 38.8 years are two factors significantly associated with HIV transmission clustering. The HIV-1 partial *env* fragment length-derived sequences were able to detect clusters of transmission contributing to the persistence of the HIV epidemic in Quebec. Although less sensitive than the sequencing of partial *env* fragments, the V3-derived sequences were able to identify HIV TCs with moderate agreement. The latter tools (V3 sequence) may be useful for short-term assessment of nascent HIV-1 transmission clustering in support of the existing methods in screening purposes.

## Sequence Data

All HIV-1 envelope nucleotide sequences for partial fragment sequences were deposited and are available in the GenBank Sequence Database (NCBI) under accession numbers KY946519 to KY946713.
